# Modeling the stimulation by glutathione of the steady state kinetics of an adenosine triphosphate binding cassette transporter

**DOI:** 10.1002/pro.4250

**Published:** 2021-12-21

**Authors:** Chengcheng Fan, Douglas C. Rees

**Affiliations:** ^1^ Division of Chemistry and Chemical Engineering Howard Hughes Medical Institute, MC 114‐96, California Institute of Technology Pasadena California USA; ^2^ Present address: Division of Biology and Biological Engineering, MC 114‐96 California Institute of Technology Pasadena California USA

**Keywords:** ABC transporter, ATPase activity, glutathione, Michaelis–Menten kinetics, nonessential activator model

## Abstract

We report the steady state ATPase activities of the ATP Binding Cassette (ABC) exporter *Na*Atm1 in the absence and presence of a transported substrate, oxidized glutathione (GSSG), in detergent, nanodiscs, and proteoliposomes. The steady state kinetic data were fit to the “nonessential activator model” where the basal ATPase rate of the transporter is stimulated by GSSG. The detailed kinetic parameters varied between the different reconstitution conditions, highlighting the importance of the lipid environment for *Na*Atm1 function. The increased ATPase rates in the presence of GSSG more than compensate for the modest negative cooperativity observed between MgATP and GSSG in lipid environments. These studies highlight the central role of the elusive ternary complex in accelerating the ATPase rate that is at the heart of coupling mechanism between substrate transport and ATP hydrolysis.

## 1 INTRODUCTION

The ability of substrates to stimulate the ATPase activity of ATP binding cassette (ABC) transporters has been utilized to identify potential substrates transported by members of this ubiquitous family.^1^, ^2^, ^3^, ^4^ Despite the practical significance of this approach for identifying the substrates of ABC exporters, more detailed kinetic characterizations of the ATPase activity as a function of the concentrations of both MgATP and transported substrate have not, to our knowledge, been reported. Such measurements could provide insight into the coupling between substrate binding and ATPase activity that is central to the mechanism of substrate translocation by ABC transporters.^5^, ^6^, ^7^, ^8^, ^9^ We accordingly conducted such studies for the bacterial ABC exporter from *Novosphingobium aromaticivorans* (*Na*Atm1), a homolog of the ABC transporter of mitochondria 1 (Atm1) family of ABC exporters.^10^, ^11^, ^12^

We have previously established that oxidized glutathione (GSSG) can be transported by *Na*Atm1.^13^ Together with structural analyses that have captured multiple conformational states, a framework for an alternating access transport cycle has been defined, with the interconversion between states coupled to the binding and hydrolysis of ATP.^13^, ^14^ These studies have established that ATP or related nucleotides are required for the formation of outward‐facing conformations by *Na*Atm1. In contrast, GSSG has only been found associated with the inward‐facing conformational state. MgATP hydrolysis requires the formation of a closed dimer interface between the two nucleotide binding domains (NBDs), with two nucleotides sandwiched between catalytic groups contributed by both domains.^15^ The catalytically relevant juxtaposition of dimerized‐NBDs is associated with the outward‐facing conformation. The preferential binding of GSSG to the inward‐facing conformation while the ATPase activity requires the outward‐facing conformation poses a paradox since it would seemingly predict that the transported substrate should inhibit the ATPase activity rather than stimulate it, as is observed. This prediction reflects the expectation that the transported substrate should thermodynamically stabilize the inward‐facing conformation that is expected to be the ATPase inactive state. As a first step to resolving this paradox, we characterized the dependence of the steady state ATPase kinetics on the concentrations of MgATP and the transported substrate, oxidized glutathione (GSSG), for the ABC exporter *Na*Atm1.

## 2 RESULTS AND DISCUSSION

### 2.1 ATPase activities

The dependence of the ATPase activity on the concentration of GSSG was measured for *Na*Atm1 reconstituted under three different conditions:

- a detergent mixture composed of 0.05% DDM and 0.05% C12E8 used in the crystallization studies.^13^, ^14^
- nanodiscs composed of the membrane scaffolding protein MSP1D1 and the phospholipid 1‐palmitoyl‐2‐ oleoyl‐glycero‐3‐phosphocholine (POPC).^16^
- proteoliposomes composed of *E.coli* polar lipids and 1,2‐dioleoyl‐sn‐glycero‐3‐phosphocholine (DOPC).^17^

**FIGURE 1:**
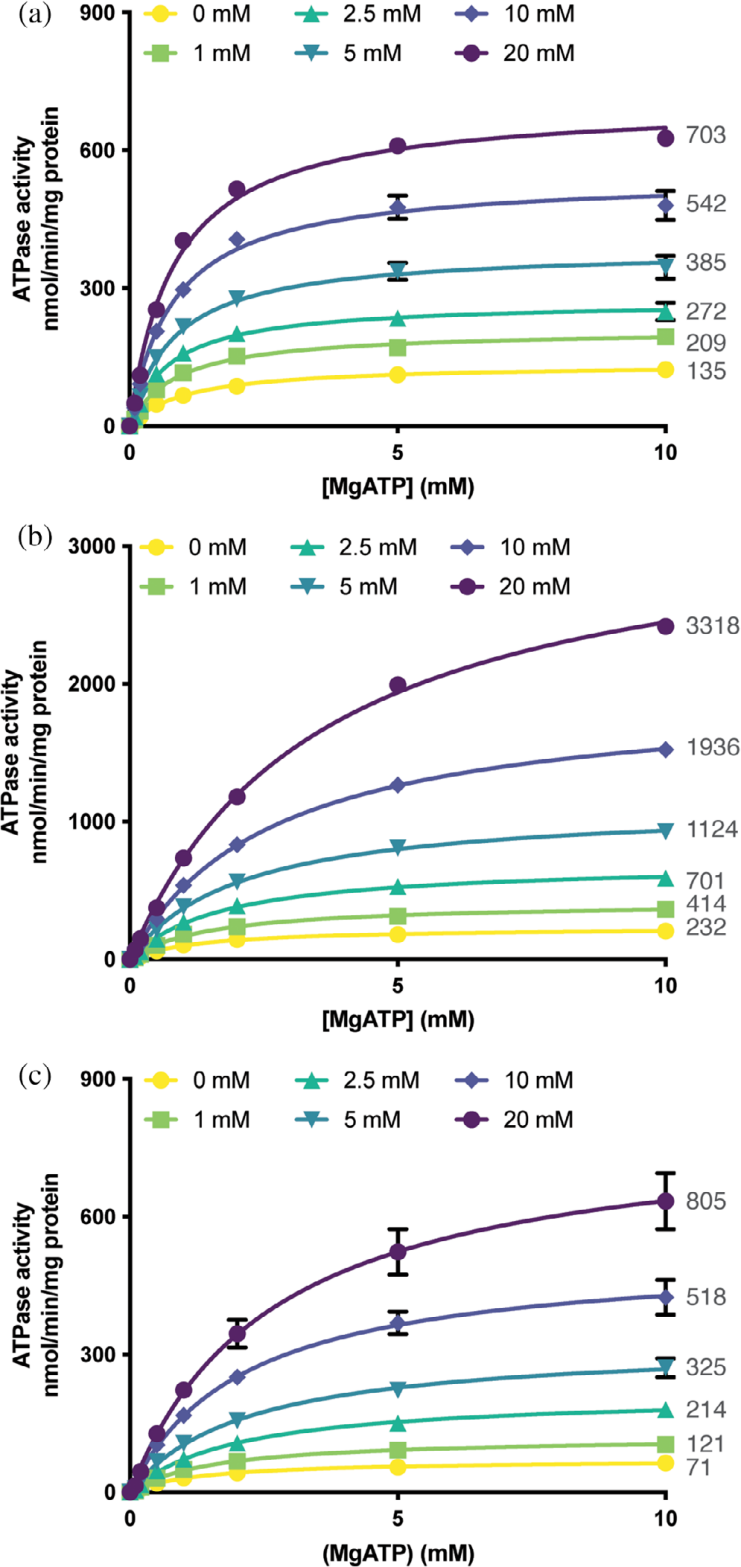
Fit of the ATPase activities of *Na*Atm1 to the Michaelis–Menten kinetic model. ATPase activities of *Na*Atm1 as a function of (MgATP) in (a) detergent (DDM/C12E8), (b) nanodiscs, and (c) proteoliposomes, with stimulations by GSSG at various concentrations. ATPase activities of *Na*Atm1 in detergent and PLS were measured six times, and three times in nanodiscs, all with distinct samples. All of the extrapolated *V*_max_ values are shown next to each GSSG concentration curve. Error bars represent the standard error of the mean for the replicates

The ATPase rates were measured in sextuplicate for the detergent and proteoliposome samples, and triplicate for the nanodisc sample under varying concentrations of MgATP (8 concentrations: 0, 0.1, 0.2, 0.5, 1, 2, 5, and 10 mM) and GSSG (6 concentrations, 0, 1, 2.5, 5, 10, and 20 mM). The ATPase rate was measured by quantifying the amount of inorganic phosphate (Pi) released upon ATP hydrolysis over a 15‐min period using a molybdate based colorimetric assay.^18^ For a given GSSG concentration, the dependence of the ATPase activity on (MgATP) was modeled by a Michaelis–Menten (hyperbolic) equation (Figure 1), yielding *k*_cat_ and Km for ATP hydrolysis as a function of GSSG concentration (Table S1). In the absence of GSSG, the values of *k*_cat_ characterizing the basal (uncoupled) ATPase rate were determined to be 18.0 ± 0.4, 30.9 ± 0.7, and 9.5 ± 0.4 min^−1^ in detergent, nanodiscs, and proteoliposomes, respectively. As the orientation effect in proteoliposomes was not taken into account, the actual ATPase rate in proteoliposomes may be ~2 times of the measured activity, or ~19 min^−1^. In each system, GSSG was observed to stimulate the ATPase activity of *Na*Atm1 in a concentration dependent fashion (Figure 1 and Table S1). The magnitude of the stimulation was dependent on the reconstitution conditions, as the *k*_cat_s in 20 mM GSSG were increased above the basal rate in the absence of GSSG by factors of ~5, 14, and 11 (Table S1a), in detergent, nanodiscs, and proteoliposomes, respectively. Less pronounced, but statistically significant changes in Km were also observed between 0 and 20 mM GSSG, corresponding to a decrease of ~20% in detergent, and increases of 2.7 times and 1.9 times for nanodiscs and proteoliposomes, respectively (Table S1b).

### 2.2 Basic nonessential activator kinetic model

**FIGURE 2:**
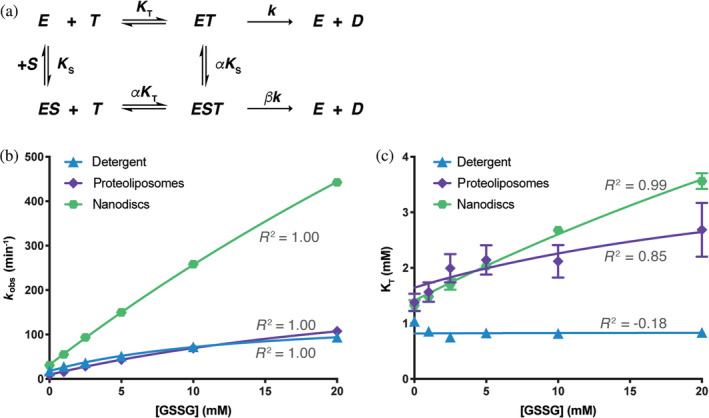
Nonessential activator model of *Na*Atm1 ATPase kinetics. (a) Schematic of the nonessential activator kinetic model for the ATPase activities of *Na*Atm1. Fits of (b) the apparent rate constant, *k*_obs_, for ATP hydrolysis (Equation (2)) and (c) the Michaelis–Menten constant, *K*_T_, of MgATP binding (Equation (3)) as a function of (GSSG) based on the experimentally derived parameters (Table 1) for the nonessential activator model for *Na*Atm1 in detergent, nanodics, and proteoliposomes, respectively. In these schemes, *E* = *Na*Atm1, *T* = MgATP, *D* = ADP, *S* = GSSG, *K*_T_ = binding constant for MgATP, *K*_S_ = binding constants for GSSG, *k* = rate constant for MgATP hydrolysis, *α* = interaction factor of how ATP binding influences GSSG binding and vice versa, and *β* = acceleration factor for ATP hydrolysis with bound GSSG, Error bars represent the experimentally observed standard deviations

To model this data, we used a nonessential activator model (Figure 2a), a steady‐state, equilibrium binding model where the transported substrate GSSG is an activator that stimulates the ATPase rate above the basal level.^19^, ^20^ The key kinetic parameters in this model are:

- *K*_T_, the Michaelis binding constant for MgATP,
- *K*_S_, the Michaelis binding constant for the transported substrate, GSSG, which is also an activator of the ATPase rate,
- *α*, the interaction factor for how binding of MgATP influences the binding of GSSG (and vice versa); *α* < 1 or > 1 denote positive and negative cooperativity, respectively,
- *k*, the basal rate constant for MgATP hydrolysis in the absence of GSSG, and
- *β*, the acceleration factor for MgATP hydrolysis with bound GSSG.

In this basic model, the ATPase sites are treated as independent since the dependence of the ATPase rate on ATP is reasonably well approximated by the hyperbolic Michaelis–Menten equation, except possibly at the lowest concentrations of ATP where some evidence for cooperativity was observed. For this scheme, expressions for the overall velocity, *k*_obs_ and *K*_T_^app^, may be derived (where *E*_T_ denotes the total concentration of transporter).

(1) $$v=\frac{E_{T}k\left\{\frac{\left[T\right]}{K_{T}}+\beta \left(\frac{\left[S\right]\left[T\right]}{\alpha K_{S}K_{T}}\right)\right\}}{1+\frac{\left[T\right]}{K_{T}}+\frac{\left[S\right]}{K_{S}}+\frac{\left[S\right]\left[T\right]}{{\mathrm{\alpha K}}_{S}K_{T}}}.$$

(2) $$k_{\mathrm{obs}}=k\frac{\left(1+\beta \frac{\left[S\right]}{\alpha K_{S}}\right)}{\left(1+\frac{\left[S\right]}{{\mathrm{\alpha K}}_{S}}\right)}.$$

(3) $$K_{T}^{\mathrm{app}}=K_{T}\frac{\left(1+\frac{\left[S\right]}{K_{S}}\right)}{\left(1+\frac{\left[S\right]}{{\mathrm{\alpha K}}_{S}}\right)}.$$

The parameters (*k*, *K*_T_, *K*_S_, *α*, and *β*) of the nonessential activator model (Equation (1), Table 1) were fit against the 48 measured ATPase rates as a function of ATP and GSSG concentrations in detergent, nanodiscs, and proteoliposomes. The basal turnover rates observed for saturating MgATP in the absence of GSSG, k, were determined to be 17.6 min^−1^ in detergent, 32 min^−1^ in nanodiscs and 9 min^−1^ in proteoliposomes (Table 1), with the binding affinities of MgATP, *K*_T_), measured as 0.82 mM in detergent, 1.41 mM in nanodiscs and 1.6 mM in proteoliposomes. The determined values for K_S_, the binding constant of GSSG, were found to be ~10 mM under all reconstitution conditions. Extrapolating to saturating concentrations of GSSG, the acceleration factors *β* were determined to be 8.3, 77, and 29 in detergent, nanodiscs, and proteoliposomes, respectively (Table 1). With these values for the parameters of the nonessential activator model, the experimental values of *k*_cat_ and Km as a function of (GSSG) were fit reasonably well (Figure 2bc), as were the fit of the individual ATPase measures as a function of MgATP and GSSG concentrations (Figure S1).

**TABLE 1:** Kinetic parameters of the nonessential activator model

Parameters	Detergent	Nanodiscs	Proteoliposomes
*k*_obs_ (min^−1^)	17.58 ± 0.75	31.86 ± 1.04	9.49 ± 1.26
*K*_T_ (mM)	0.82 ± 0.07	1.41 ± 0.08	1.64 ± 0.34
*K*_S_ (mM)	13.34 ± 2.20	9.65 ± 0.74	12.79 ± 4.09
Αlpha (*α*)	1.03 ± 0.20	10.05 ± 1.73	2.64 ± 1.26
Beta (*β*)	8.30 ± 0.46	76.60 ± 8.68	28.69 ± 6.30

*Note*: Parameters calculated with the nonessential activator model shown in Figure 2 for the ATPase activities of *Na*Atm1 in detergent, nanodiscs, and proteoliposomes. The *R* ^2^ values are in the range of 0.94 to 0.99 for the measurements in detergent, 0.99–1.00 for the measurements in nanodiscs and 0.91–0.95 for the measurements in proteoliposomes. Parameters are tabulated to two decimal places to accurately reproduce the calculations depicted in Figures 2 and S1.

The results of this analysis demonstrate the ATPase kinetics are dependent on the lipid environment, explored in this work as detergent, nanodiscs, and proteoliposomes, and that the basal ATPase rates are stimulated by GSSG under these reconstitution conditions. While the primary influence of GSSG is to accelerate *k*_cat_, the Km values for ATP are also impacted. These results provide the opportunity to explore the binding interactions between ATP and GSSG, as reflected in the cooperativity parameter α. While little cooperativity is evident in detergent (*α* ~ 1.025), in the lipid environment provided by reconstitution into nanodiscs and proteoliposomes, evidence for modest negative cooperativity is observed with *α* ~ 10 and 3, respectively. These trends are similarly reflected in the increases in *K*_T_, the Michaelis constant for ATP, between 0 and 20 mM observed for nanodiscs and proteoliposomes.

For an allosteric system described by a classical Monod–Wyman–Changeux model,^21^ a ligand that preferentially binds to the inactive conformation of a two‐state system will function as an inhibitor. GSSG appears to bind preferentially to the inward‐facing conformation of *Na*Atm1, while the catalytically competent conformation for ATP hydrolysis is the outward‐facing conformation. Based on an equilibrium binding model, it would be anticipated that GSSG and MgATP should exhibit negative cooperativity towards each other. This expectation is reflected in the *α* > 1 values for *Na*Atm1 reconstituted in a lipid environment, corresponding to the increases in *K*_T_ between 0 and 20 mM GSSG under those conditions. Nevertheless, GSSG significantly stimulates the ATPase rate, which suggests that the kinetics of forming the outward‐facing conformations of *Na*Atm1 differ in important ways between the binary (with MgATP) and ternary (with MgATP and GSSG) complexes. A key mechanistic question is why the ternary complex has an accelerated ATPase rate. Structural studies have yet to provide any insights into this question as no structures of this ternary complex have been determined for an ABC exporter. Understanding how this species promotes ATP hydrolysis relative to the binary complex is at the heart of the coupling mechanism and emphasizes the importance of characterizing the structure and dynamics of this elusive transporter‐ATP‐substrate ternary state.

## 3 MATERIAL AND METHODS

### 3.1 Protein expression, purification, and reconstitution

The over‐expression of *Na*Atm1 (Addgene catalog number 78308) was achieved with *Escherichia coli* BL21‐gold (DE3) cells (Agilent Technologies) using ZYM‐5052 autoinduction media as described previously.^13^ Cells were harvested by centrifugation and stored at −80°C until use.

Protein purification was carried out as previously described.^14^ Briefly, frozen cell pellets were resuspended in lysis buffer containing 100 mM NaCl, 20 mM Tris, pH 7.5, 40 mM imidazole, pH 7.5, 10 mM MgCl_2_, 0.5% (wt/vol) n‐dodecyl‐β‐d‐maltopyranoside (DDM) (Anatrace), and 0.5% (wt/vol) octaethylene glycol monododecyl ether (C12E8) (Anatrace) in the presence of lysozyme, DNase, and protease inhibitor tablet. Resuspended cells were solubilized by stirring for 3 h at 4°C. The lysate was ultracentrifuged at 113,000 x *g* for 45 min at 4°C to remove unlysed cells and cell debris. The supernatant was loaded onto a prewashed NiNTA column. NiNTA wash buffer contained 100 mM NaCl, 20 mM Tris, pH 7.5, 50 mM imidazole, pH 7.5, 0.05% DDM and 0.05% C12E8 and elution buffer contains 350 mM imidazole instead. The eluted sample was then subjected to size exclusion chromatography using HiLoad 16/60 Superdex 200 (GE Healthcare) with buffer containing 100 mM NaCl, 20 mM Tris, pH 7.5, 0.05% DDM, and 0.05% C12E8. Peak fractions were collected and concentrated with Amicon Ultra 15 concentrator (Millipore) (MW 100 kDa) to ~20 mg/ml.

*Na*Atm1 nanodiscs in membrane scaffolding protein and proteoliposomes were prepared as described previously.^14^ Additional BioBeads were added at 50 mg/ml to ensure the complete removed of detergent in both reconstitutions. The protein concentration in the nanodisc preparations includes contributions from both the transporter and the scaffold protein; for calculating the *k*_cat_ values, it is assumed that the ratio of scaffold protein to transporter in the nanodiscs is 2:1 based on the EM density. Consequently, the transporter concentration was calculated as 135/(135 + 2 × 25) = 0.73 of the total protein concentration based on molecular weights of 25 kDa and 135 kDa for the scaffold protein and *Na*Atm1, respectively.

### 3.2 ATPase assay

The ATPase activities were measured by the molybdate based phosphate quantification method^18^ as described previously.^14^ All reactions were carried out at 37°C in 250 μl scale. The reaction mixture contained a final *Na*Atm1 concentration of 0.05 mg/ml in 100 mM NaCl and 20 mM Tris, pH 7.5, with varying concentrations of MgATP and GSSG. For each reaction, 50 μl of reaction mixture was taken every 5 min for 4 times and subsequently mixed with 50 μl of 12% SDS, 100 μl of ascorbic acid/molybdate mix, and 150 μl of citric acid/arsenite/acetic acid solution before reading with a Tecan plate reader at 850 nm. Reactions in detergent and proteoliposomes were done in sextuplicates and reactions in nanodiscs were done in triplicates. The measurements were plotted against time to obtain the ATPase rates. The final rates were fitted into Michaelis–Menten kinetics or the nonessential activator model in Mathematica and Prism 9. The graphs were plotted in Prism 9. The unweighted least regression resulted in *R* ^2^ values of 0.98, 1.00, and 0.95 for the measurements in detergent, nanodiscs, and proteoliposomes, respectively.

## CONFLICT OF INTEREST

The authors declare no competing interests.

## AUTHOR CONTRIBUTIONS

**Chengcheng Fan:** Conceptualization (equal); data curation (lead); formal analysis (equal); investigation (lead); methodology (lead); validation (lead); visualization (lead); writing – original draft (lead); writing – review and editing (equal). **Douglas C. Rees:** Conceptualization (equal); formal analysis (equal); funding acquisition (lead); project administration (lead); supervision (lead); writing – review and editing (equal).

## Supporting information


Data S1: Supporting Information.
