# Supplementary material for: Modeling the stimulation by glutathione of the steady state kinetics of an adenosine triphosphate binding cassette transporter
Source: Protein Sci. 2021 Dec 21;31(3):752–7. doi: 10.1002/pro.4250 (PMC8862428; doi:10.1002/pro.4250)
Supplement: Supplementary file 1 — Data S1: Supporting Information. [file PRO-31--s001.pdf]

Supplementary materials

Modeling the stimulation by glutathione of the steady state kinetics of an adenosine triphosphate binding cassette transporter

Chengcheng Fan[1,2] and Douglas C. Rees[1,*]

[1]Division of Chemistry and Chemical Engineering, Howard Hughes Medical Institute, MC 114-96, California Institute of Technology, Pasadena, CA 91125, USA

[2]Present address: Division of Biology and Biological Engineering, MC 114-96, California Institute of Technology, Pasadena, CA 91125, USA

*Correspondence
Douglas C. Rees
Email: dcrees@caltech.edu
Office telephone: (626) 395-8393
Fax: (626) 744-9524
Address: 1200 E. California Blvd., MC 114-96, Pasadena, CA 91125

Running title (49 characters): GSSG Stimulation of the ATPase kinetics of *Na*Atm1

Supplementary materials pages: 3 (including title page, 1 figure and 1 table)

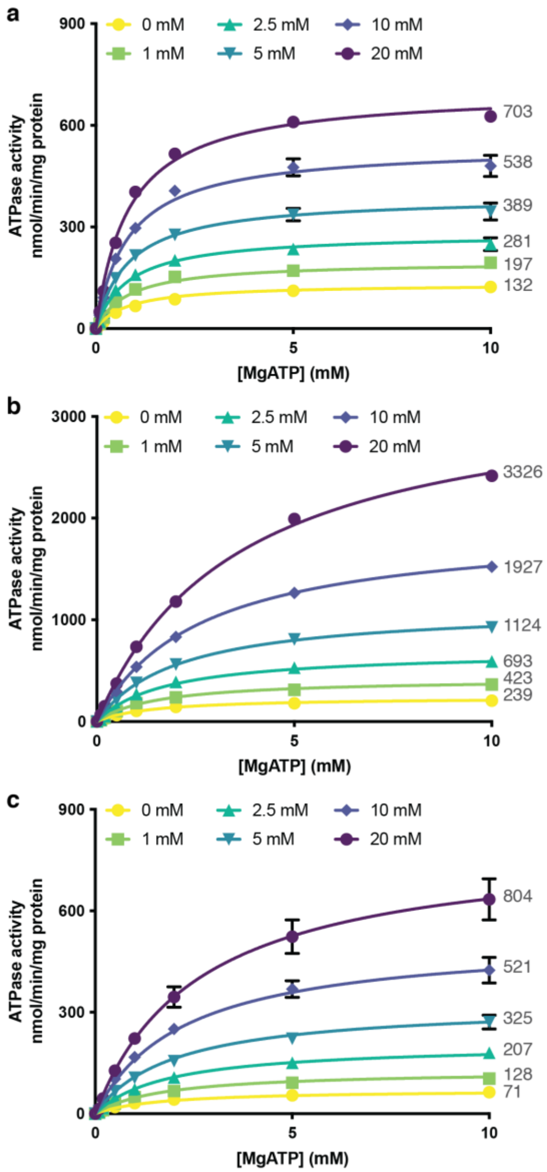

Figure S1. Fit of the ATPase activities of *Na*Atm1 to the non-essential activator model. The ATPase activities of *Na*Atm1 in (**a**) detergent (DDM/C12E8), (**b**) nanodiscs, and (**c**) proteoliposomes as a function of (MgATP) and (GSSG) were calculated from Eq. 1 of the non-essential activator model using the kinetic parameters in Table 1. ATPase activities of *Na*Atm1 in detergent and PLS were measured six times, and three times in nanodiscs, all with distinct samples. All of the extrapolated V_{max} values are shown next to each GSSG concentration curve. Error bars represent the standard error of the mean for the replicates.

a

GSSG (mM)	Detergent	Nanodiscs	Proteoliposomes
**0**	18.0 ± 0.4	30.9 ± 0.7	9.5 ± 0.4
**1**	27.9 ± 0.7	55 ± 1	16.1 ± 0.6
**2.5**	36 ± 1	93 ± 2	29 ± 1
**5**	51 ± 2	150 ± 2	43 ± 2
**10**	72 ± 2	258 ± 3	69 ± 3
**20**	94 ± 1	442 ± 7	107 ± 8

b

GSSG (mM)	Detergent	Nanodiscs	Proteoliposomes
**0**	1.04 ± 0.07	1.32 ± 0.10	1.4 ± 0.2
**1**	0.86 ± 0.07	1.48 ± 0.08	1.6 ± 0.2
**2.5**	0.75 ± 0.09	1.70 ± 0.10	2.0 ± 0.3
**5**	0.83 ± 0.08	2.03 ± 0.09	2.1 ± 0.3
**10**	0.82 ± 0.08	2.70 ± 0.07	2.1 ± 0.3
**20**	0.84 ± 0.04	3.6 ± 0.1	2.7 ± 0.5

Table S1. Calculated Michaelis-Menten parameters. (**a**) Apparent rate constants for ATP hydrolysis and (**b**) apparent binding affinity of MgATP at different concentration of GSSG. The R^2 values are in the range of 0.94 to 0.99 for the measurements in detergent, 0.99 to 1.00 for the measurements in nanodiscs and 0.91 to 0.96 for the measurements in proteoliposomes.
